# Pulmonary artery pseudoaneurysm: an unsuspected cause of haemoptysis in post-COVID fungal infection of the chest

**DOI:** 10.1259/bjrcr.20210198

**Published:** 2022-03-04

**Authors:** Samvid Kotia, Shrishail Adke, Padma Vikram Badhe, Krantikumar Rathod

**Affiliations:** 1 Department of Radiodiagnosis, Seth GS Medical College and KEM Hospital, Parel, Mumbai, Maharashtra, India

## Abstract

Pulmonary Artery Pseudoaneurysm (PAP) is a rare but potentially fatal cause of haemoptysis, which often remains unsuspected by both clinicians and radiologists.

Traditionally, infections like tuberculosis and bacterial endocarditis have been associated with the development of PAPs. However, additional causative factors like trauma, neoplasia, pulmonary hypertension and vasculitis are also to be considered.

With the advent of the novel Coronavirus (COVID-19), attempts have been undertaken to study its multisystem implications. Also, a strong correlation has also been established between COVID-19 and fungal infestation of the paranasal sinuses and lung parenchyma. Hence, PAP should be suspected in post-COVID patients who develop new-onset haemoptysis or new focal consolidation on imaging.

Imaging investigations like chest radiograph, CT chest, and CT Pulmonary Angiography help in the establishment of a diagnosis and assessment of the relevant anatomy, which aid in the classification of the PAP. Management strategies include endovascular treatment, surgical resection or conservative approach in form of prolonged antimicrobial therapy. Interventional radiological procedures like endovascular embolisation are especially useful in vitally unstable cases of massive haemoptysis who are poor surgical candidates.

Our case highlights the unique presentation of pulmonary arterial pseudoaneurysm induced by a post-COVID-19 fungal infection.

## Case presentation

A 30-year-old male with a history of COVID-19 pneumonia two months back, presented with a new onset cough with haemoptysis since two weeks. He initially had streaky intermittent haemoptysis. The patient then developed progressive breathlessness and had five episodes of massive haemoptysis.

## Investigations

**Figure 1.**
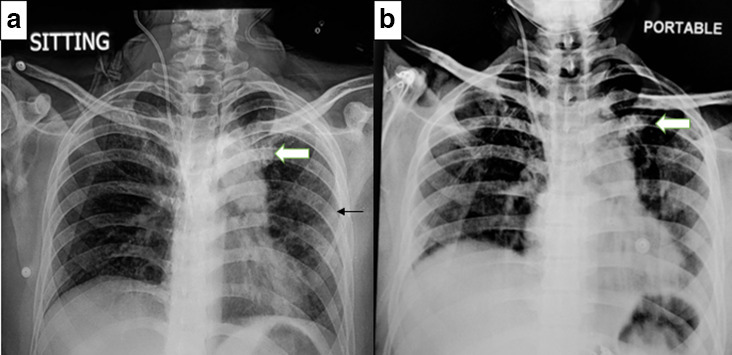
Frontal chest radiograph taken on Day 1 of admission (**a**) shows a patchy area of fibrosis in the left upper zone (white block arrow) with fine linear opacities in the left hemithorax with subpleural predominance (black arrow). Serial radiograph taken on Day 4 of admission (**b**) shows minimal increase in the size of the lesion (white block arrow).

A frontal chest radiograph was performed (Figure 1) which shows findings consistent with post Covid fibrosis in the form of subpleural linear fibrotic opacities in the left hemithorax with a patchy area of fibrosis in the left upper lobe.

**Figure 2.**
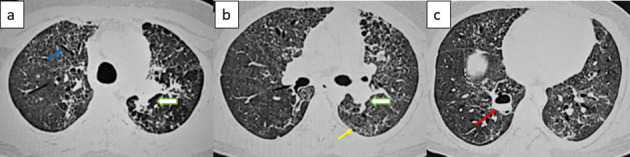
Axial HRCT chest image (**a**) reveal dilated bronchi (blue arrow); (**b**) inter- and intraseptal thickening predominantly in subpleural region(yellow arrow) and (**c**) small-sized thin-walled cavity with air-fluid level (red arrow). Focal patchy area of parenchymal opacification (white block arrows) was seen in the apicoposterior segment of left upper lobe.[Figure 2(b)]

High-Resolution Computed Tomography (HRCT) Chest (Figure 2) performed subsequently shows post-Covid fibrosis in form of fibrotic scarring and traction bronchiectasis with a fibrotic patch in the apico-posterior segment of the left upper lobe. A small thin-walled cavity with an air-fluid level was seen in the medial segment of the right lower lobe.

In view of imaging findings of a lower lobe cavity in a setting of post-infective sequelae, fungal infection was suspected, hence Serum galactomannan was done that turned out to be positive.

Based on imaging and laboratory correlation, a diagnosis of post-covid fungal infection was made and Amphotericin therapy was initiated.

**Figure 3.**
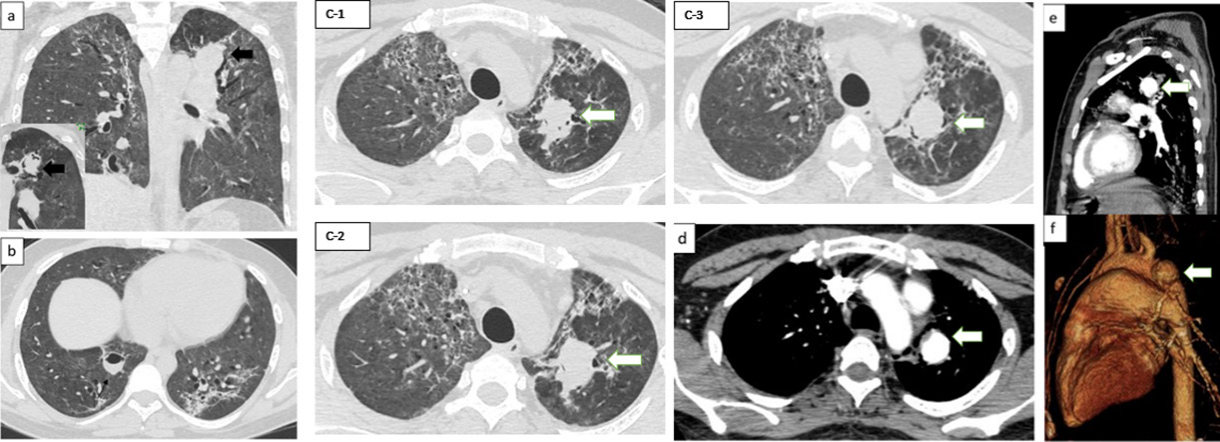
Pulmonary artery pseudoaneurysm. coronal (**a**) and serial axial (c-1,c-2,c-3) HRCT images show a focal zone of consolidation (white block arrows) in the left upper lobe. It is also seen abutting the left upper lobe bronchus on MiniP coronal image (inlet image in Figure 3a). Axial image of a lower section (**b**) shows partial resolution in the size of the fungal cavity. Axial sections of the pulmonary angiogram (**d**) Sagittal reformatted MIP image (**e**) and Sagittal 3D VRT image (**f**) shows the pseudoaneurysm communicating with the segmental branch of the left upper lobar artery.

A repeat HRCT chest was performed 20 days later, after the patient had multiple episodes of massive haemoptysis. It showed partial resolution of the previously seen cavity (Figure 3b) and the development of a focal zone of consolidation in the left upper lobe at the site of the previous fibrotic patch (Figure 3a, b and c). This lesion was abutting the left upper lobe pulmonary vessels (Figure 3f showing sagittal reformatted MIP-Maximum Intensity Projection image; 3f showing sagittal 3D VRT – Volume Rendering Technique image). It was also seen adjacent to the left upper lobe bronchus (Figure 3a-inset image). Hence, a suspicion of pseudoaneurysm causing haemoptysis was raised.

A contrast-enhanced CT (CECT) chest with Computed Tomography Pulmonary Angiography (CTPA) was performed for diagnostic confirmation (Figure 3d, e and f).

It shows the presence of a focal well-defined thin-walled contrast-filled outpouching arising from the left upper lobe branch of the pulmonary artery measuring 2.2 × 2 × 2 cm (Anteroposterior x Transverse x Craniocaudal) suggestive of pseudoaneurysm. There were no other pseudoaneurysms.

## Differential diagnosis

Persistence of respiratory symptoms or development of new respiratory symptoms has been observed in half of the patients, which had a history of COVID-19, especially those who required oxygen therapy.^1^ In such patients, the appearance of a new focal consolidation should prompt the radiologist to consider the following differential diagnosis:

1. Pulmonary thromboembolism

CT Pulmonary angiography with routine HRCT chest should be considered when there is clinical suspicion for PE or when Well’s score is high (>6). Imaging findings include consolidation, bronchial dilatation within involved portions of the lungs, and a filling defect involving a segmental or main trunk of the pulmonary arteries.

2. Superimposed pneumonia^2^

Superimposed pneumonia is seen in 10% of hospitalized patients with post-covid status. Patients with COVID-19 and ARDS may die owing to superimposed bacterial or fungal infection. Hence, chest imaging and lower respiratory cultures should be considered whenever secondary respiratory worsening occurs in a post-Covid patient. Lobar consolidation at chest imaging may reflect superimposed bacterial pneumonia.

3. Post covid fibrosis

The most common findings are parenchymal bands, irregular interfaces, coarse reticular pattern and bronchial dilation.^3^ Tinted sign that has been described as a decrease in the density and increase in the extent of ground-glass opacification is seen in a few patients with fibrotic nodules. It is usually seen three to four weeks after the acute phase and is related to broncho-vascular bundle distortion.

4. Arterial pseudoaneurysm

Although uncommon, as in our case, pulmonary artery pseudoaneurysm is an important cause of haemoptysis. The mortality rate associated with the rupture of a PAP is as high as 50%; death is secondary to aspiration and asphyxia after intrapulmonary haemorrhage.^4^ Imaging findings include new-onset consolidation near previous areas of consolidation or a vessel seen reaching up to an area of consolidation. In all cases of suspected pseudoaneurysm, CT pulmonary angiography should be performed to identify and localise the bleeding sites amongst the pulmonary, bronchial and non-bronchial collateral circulations.

## Treatment

**Figure 4.**
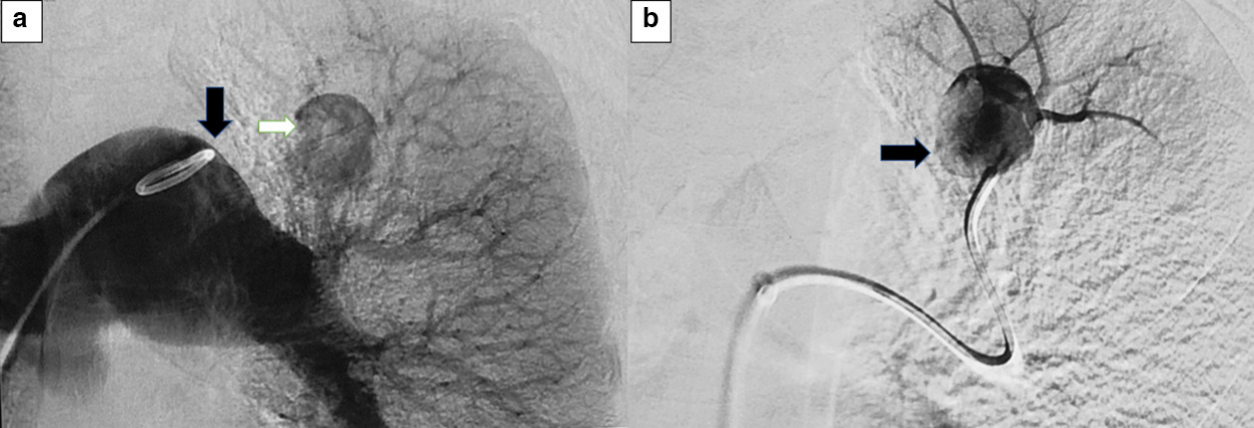
(**a**) Non-selective angiogram performed with a pigtail catheter (Black arrow) positioned at the bifurcation of the right and left pulmonary artery shows contrast material filling the PAP (white arrow) with the left upper lobe segmental artery as the feeding vessel. (**b**) Selective catheterisation of the left upper lobe artery was performed and contrast was injected to confirm the vascular supply of the pseudoaneurysm.

**Figure 5.**
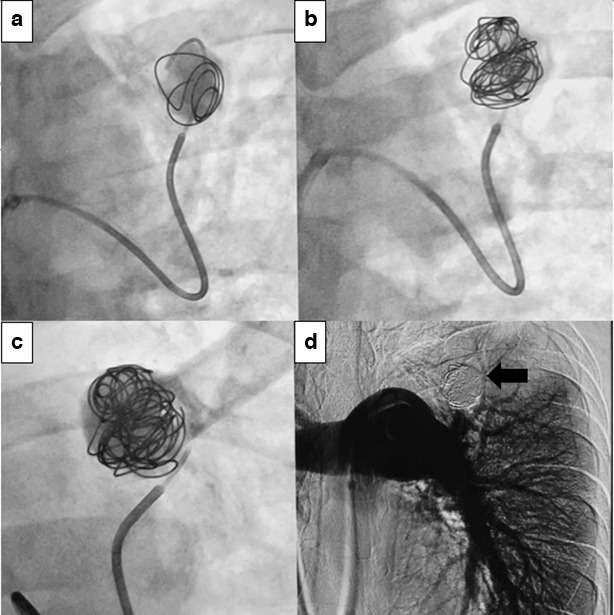
(**a**) to (**c**) shows embolisation using multiple pushable coils. Figure (**d**) shows non-opacification of the previously seen pseudoaneurysm (black arrow) suggestive of successful embolisation.

Using all aseptic precautions and under local anaesthesia, Right IJV access was obtained using a 6F Balkin sheath. Pulmonary angiography was performed with a pigtail catheter positioned at the bifurcation, which revealed a left upper lobe pulmonary artery pseudoaneurysm.(Figure 4) Super-selective catheterisation was done using co-axial system of 5F Sim-1 catheter and 2.7F Progreat microcatheter via right IJV access. Mechanical embolisation was performed using multiple push-able micro-coils. Platinum coils of size 0.018 with synthetic fibre coating were used. These are inexpensive, provide excellent vessel occlusion and the synthetic coating increases thrombogenicity and promote clot formation. Two 14 mm × 10 cm coils were used for the initial framing of the aneurysm using the “push” technique. After their successful deployment, two 14 mm × 8 cm coils were used for packing.(Figure 5) The neck of the pseudoaneurysm and dysplastic supplying branch were treated by glue embolisation using N-butyl cyanoacrylate (Endocryl, India).

Post-procedure repeat angiogram revealed complete occlusion of the aneurysm.

## Outcome and follow-up

After the endovascular therapy, the patient had a resolution of cough and breathlessness. He did not have any further episodes of massive haemoptysis. He had a single episode of streaky haemoptysis on Day 3 post-procedure; however, the rest of his hospital stay was uneventful.

The patient came for a follow-up three months after the procedure. He did not have any haemoptysis episodes during this period.

## Discussion

Massive haemoptysis is defined as pulmonary haemorrhage with expectoration of a volume greater than 300 ml during a 24-h period. The mortality is more than 50% in untreated cases.^5^ Although bronchial arteries and non-bronchial systemic collaterals are more common causes, pulmonary artery as an aetiology is seen in upto 10% of cases of massive haemoptysis.^6^ Pseudoaneurysm involving the pulmonary artery occurs when there is a contained rupture of the pulmonary artery and is seen in 5–11% of patients who underwent bronchial or pulmonary angiography. Early diagnosis is imperative to prevent life-threatening haemoptysis for which CT Pulmonary Angiography has become the diagnostic method of choice. CT Pulmonary Angiography is also essential in differentiating it from other causes of focal consolidation like nodular masses of endobronchial origin and hence prevents unnecessary biopsies or surgeries.^7^

The most common cause of acquired PAPs is infection. The PAPs associated with tuberculosis (also known as Rasmussen aneurysm) is due to contiguous spread from the tuberculous cavity. There is the destruction of the outer vessel wall caused by the tissue destruction from the expanding cavity which finally weakens the vessel wall and leads to pseudoaneurysm formation.^8^ A similar mechanism of pathogenesis is seen in cases of bacterial pneumonia. In cases of PAP developing after COVID-19, pulmonary vasculitis and severe inflammation are considered the most accepted mechanism. This occurs mainly due to upregulation of pro-inflammatory cytokines, complement activation, endothelial damage/dysfunction. Other postulated mechanisms for small vessel damage is the formation of immune-thrombotic clots. This phenomenon is triggered by the viral RNA molecules and leads to immune cell infiltration into the pulmonary vasculature. These immune cells along with tissue factor and inflammatory cytokines lead to immunothrombus formation which further weakens the vessel wall.

PAPs that are not directly related to lung consolidation may occur due to causes like septic emboli. Here, the destruction of the vessel wall starts in the lumen (due to endovascular seeding) and then progresses to the outer wall.^8^

Another major cause of PAPs is a traumatic injury to the pulmonary vasculature by iatrogenic [intercostal drainage tube (ICD) insertion, radiofrequency ablation of lung tumours] or non-iatrogenic causes. An acute injury is more likely to result in haemorrhage surrounding the area of consolidation.

Pulmonary embolism, lung cancers and metastases may cause local pulmonary arterial wall injury and rarely may cause pseudoaneurysm formation.

Endovascular treatment is a standard technique for infection-related PAP as an emergency open thoracic procedure is associated with mortality rates as high as 43%.^9^ As infectious pseudoaneurysms can be simultaneously perfused by systemic and pulmonary circulation, their endovascular treatment includes embolisation of any bronchial, non-bronchial systemic collateral artery, or pulmonary artery feeder branch, which is according to the pattern of the blood supply.^9^

Different endovascular embolisation techniques used for PAPs include vascular occlusion devices, coils, polyvinyl alcohol particles, or absorbable gelatin particles. However, particle embolic agents are not ideal for feeding artery embolisation of PAPs because increasing pressure during the infusion of a particulate agent can cause a pseudoaneurysm to rupture. Micro-coils are the most normally employed and preferred embolic materials for feeding-branch embolisation of PAPs. According to the pattern of the blood supply from the pulmonary artery, bronchial and non-bronchial systemic collateral arteries, PAPs were classified into four groups.(Table 1)

**Table 1.** Different types of pulmonary artery PAPs and their endovascular management.^9^

TYPE	MANAGEMENT
A (seen on non-selective pulmonary angiography) Denotes patent feeding artery without significant bronchopulmonary shunting	Catheterisation of feeding branch of PAP followed by embolisation with micro-coils or glue
B (seen only on selective pulmonary angiography) Denotes stenosis of feeding artery or reversal of flow due to a bronchopulmonary shunt	Catheterisation of feeding branch of PAP followed by embolisation with micro-coils or glue
C (Not seen on pulmonary angiography; seen only on bronchial or systemic collateral angiography) Denotes stenosis of feeding artery with supply from the bronchial artery or systemic collaterals via a bronchopulmonary shunt	If the lesion is accessible percutaneously, Percutaneous injection using bovine thrombin or N-butyl cyanoacrylate If the lesion is not accessible via percutaneous route, Emergent surgery with stabilisation post wedge resection
D (Not seen on catheter angiography) Denotes stenosis of feeding artery with slow systemic arterial flow which is not detected on catheter angiography.	If the lesion is accessible via percutaneous route, Percutaneous injection using bovine thrombin or N-butyl cyanoacrylate If the lesion is not accessible via percutaneous route, Emergent surgery with stabilisation post wedge resection

## Learning points

- The appearance of a new focal consolidation in a post covid patient with haemoptysis should raise the suspicion of a PAP and a CT pulmonary angiogram should be performed.
- Once identified a CT angiography should be done to identify the bleeding sites and bleeding vessels, including bronchial non-bronchial systemic collateral arteries, and pulmonary arteries.
- Angiographic classification of the type of pseudoaneurysm helps in guiding further endovascular treatment.
